# Adjuvant Effect of Cinnamon Polyphenolic Components in Colorectal Cancer Cell Lines

**DOI:** 10.3390/ijms242216117

**Published:** 2023-11-09

**Authors:** Alessandro Palmioli, Matilde Forcella, Monica Oldani, Irene Angotti, Grazia Sacco, Paola Fusi, Cristina Airoldi

**Affiliations:** Department of Biotechnology and Biosciences, University of Milano-Bicocca, P.zza della Scienza, 2, 20126 Milan, Italy; alessandro.palmioli@unimib.it (A.P.); matilde.forcella@unimib.it (M.F.); m.oldani12@campus.unimib.it (M.O.);

**Keywords:** colorectal cancer (CRC), cinnamon metabolic profiling, flavonoids, procyanidins, cetuximab, adjuvant therapy

## Abstract

Colorectal cancer (CRC) is the second-leading cause of cancer death, with a worldwide incidence rate constantly increasing; thus, new strategies for its prevention or treatment are needed. Here, we describe the adjuvant effect of the polyphenol-enriched fractions of cinnamon, from cinnamon bark and buds, when co-administered with a potent anticancer drug, cetuximab, used for CRC therapy. The co-administration significantly reduces the cetuximab dose required for the antiproliferative activity against colorectal cancer cell line E705, which is sensitive to EGFR-targeted therapy. The anticancer activity of these cinnamon-derived fractions, whose major components (as assessed by UPLC–HRMS analysis) are procyanidins and other flavonoids, strictly correlates with their ability to induce apoptosis in cancer cell lines through ERK activation and the mitochondrial membrane potential impairment. Due to the severe side effects of cetuximab administration, our results suggest the use of nutraceuticals based on the polyphenolic fractions of cinnamon extracts as adjuvants in the therapy of CRC.

## 1. Introduction

Colorectal cancer (CRC) is the second-leading cause of cancer death and the third-most prevalent malignant tumor worldwide; its incidence rate is constantly increasing [1]. New approaches for the prevention or treatment of CRC are being constantly developed.

The ideal CRC treatment would achieve complete removal of the tumor and metastasis, which mostly requires surgical intervention [2]. For those patients with unresectable lesions or who have disease which has disseminated too much, the goal is maximum shrinkage of the tumor and suppression of further tumor spread and growth; chemotherapy is the leading strategy in these patients. However, chemotherapy has several drawbacks, including potentially lethal side effects, systemic toxicity, unsatisfying response rate, unpredictable innate and acquired resistance, and low tumor-specific selectivity [3].

The idea of molecular-targeted therapy has emerged in the last two decades. The epidermal growth factor receptor (EGFR) is a driver in many cancers and, as a consequence, is a major target in oncology. Monoclonal antibodies (mAbs), as well as small molecules like tyrosine kinase inhibitors, are used as a treatment for patients with a variety of solid tumors [4,5]. Regarding CRC, due to the mechanisms of activation in this disease, mAbs represent the elective choice. Cetuximab and panitumumab are now approved by international guidelines and act against EGFR by competitive binding with the EGFR ligand (EGF), leading to the inhibition of downstream signaling pathways involved in cell survival, proliferation, metastasis, and angiogenesis [6]. These drugs have several limitations: they are expensive; they are characterized by side effects such as severe skin toxicity (occurring in approximately 80% of patients), corneal erosion, headache, pulmonary damages, general weakness, and diarrhea [7,8,9]; and, more importantly, they have been proven to be effective in providing clinical benefit in no more than 30% of patients [10]. Such inefficacy is essentially related to primary resistance due to co-occurring mutations in EGFR downstream pathways; the most diffused and widely recognized are KRAS/NRAS and BRAF mutations [11], cumulatively observed in up to 50% of patients. But, even in KRAS/NRAS/BRAF wild-type patients, the ratio of patients who benefit from mAbs against EGFR does not increase dramatically, indicating that other mechanisms are relevant in this process and need to be uncovered.

Therefore, there is an urgent need to identify new approaches, such as the application of nutraceuticals, which are able to both elicit and expand the range of chemo-preventive actions while reducing the amount of drugs administered during a therapeutic cycle [12]. Nutritional therapy and phytotherapy have emerged as new concepts, and healing systems have quickly and widely spread in recent years. Natural products have long been regarded as one of the potential materials for developing anticancer agents. 

Cinnamon is a popular flavoring ingredient, widely used in food products. It exhibits health-beneficial properties; it has been widely used for treating blood circulation disturbances, dyspepsia, allergic disease, gastritis, diabetes, and hypertension [13] and has been reported to have neuroprotective and anti-inflammatory effects, as well as anticancer activity [14,15,16,17]. In particular, Lu and coworkers designed and synthesized a novel series of cinnamaldehyde-based aspirin derivatives endowed with anticancer activity toward CRC [18].

In the present study, the effects of hydroalcoholic extracts of bark from *Cinnamomum cassia* (CCHE) or *Cinnamomum zeylanicum* (CZHE) or buds from *Cinnamomum cassia* (BCHE)—and of the corresponding fractions enriched in polyphenols (B) or cinnamaldehyde (C)—on colorectal healthy and cancer cell lines have been evaluated. In particular, cell lines were treated for 48 h, and cell viability, apoptotic rate, and levels of P-ERK, caspase 3, and BCL2 were evaluated. These results allowed the elucidation of the anticancer activity and the potential molecular mechanism of cinnamon fractions enriched in polyphenols on human colorectal cancer cell lines.

## 2. Results

### 2.1. Characterization of Cinnamon Extract—Polyphenol-Enriched Fractions

The NMR-based metabolic profiling of hydroalcoholic extracts of bark from *Cinnamomum cassia* (CCHE) or *Cinnamomum zeylanicum* (CZHE) or buds from *Cinnamomum cassia* (BCHE) cinnamon was already reported by our group (Supplementary Material—Figure S1), together with a preliminary UPLC–HRMS-based analysis of a fraction (fraction B) of BCHE and CCHE extract enriched in polyphenol components [17]. Their chemical composition, showing the presence of polyphenols which have already been reported to have the ability to modulate the growth of tumor cell lines [19], together with the significant amount of cinnamaldehyde and congeners, whose antiproliferative activity was previously described [20,21], prompted us to investigate the potential chemopreventive and anticancer activity of the extracts and of the corresponding polyphenol- and cinnamaldehyde-enriched fractions, obtained by preparative reverse-phase (RP) C18 flash chromatography (Supplementary Material—Figures S2–S4). 

**Figure 1:**
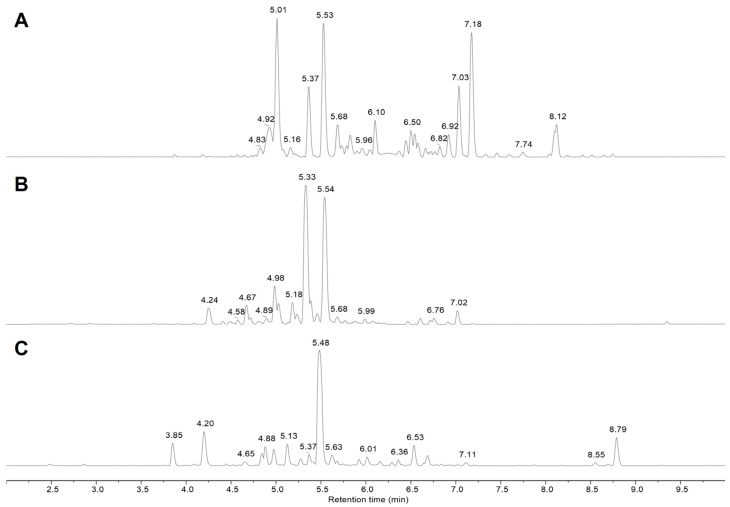
UPLC–HRMS analysis of polyphenol-enriched fractions. Base peak chromatogram (negative ionization mode) of fractions B obtained from *Cinnamomum cassia* buds (BCHE, panel **A**) and bark (CCHE, panel **B**) and *Cinnamomum zeylanicum* (CZHE, panel **C**) extracts are reported.

Figure 1 reports the UPLC–HRMS base peak chromatograms of fractions B obtained from *Cinnamomum cassia* buds (BCHE, Figure 1A) and bark (CCHE, Figure 1B) and *Cinnamomum zeylanicum* (CZHE, Figure 1C) extracts, showing significant differences among the chemical compositions of the three samples.

The detailed MS analysis of fractions B (Table 1) revealed the presence of a great amount of A- and B-type procyanidins, ranging from dimer till to heptamers, but also of monomeric flavonoids (in glycosylated form), phenyl glycosides, and chalcones. MS/MS spectra of the major compounds identified in polyphenol-enriched fractions (fraction B) obtained from *Cinnamomum cassia* buds are reported in Supplementary Material—Figure S5 as representative of the analysis performed on all three fractions B.

### 2.2. Cytotoxic Effect of Cinnamon Extracts on Colorectal Cancer Cell Lines

We evaluated the effect on the viability of different colorectal cell lines of hydroalcoholic extracts and fractions enriched in either polyphenols (B) or cinnamaldehyde (C) of bark from *Cinnamomum cassia* (CCHE) or *Cinnamomum zeylanicum* (CZHE) or buds from *Cinnamomum cassia* (BCHE). The chosen cell lines were a healthy colorectal mucosa CCD841 cell line and three colorectal cancer cell lines with peculiar molecular features. In particular, Caco-2 and E705 show no hyperactivating mutations in KRAS, NRAS, BRAF, and PIK3CA genes, with the E705 cell line carrying a silent mutation in the PIK3CA gene, whereas the SW480 cell line carries a hyperactivating mutation in exon 2 of the KRAS gene. Caco-2 and SW480 cell lines do not respond to cetuximab, while the E705 cell line is sensitive to cetuximab [22]. 

**Figure 2:**
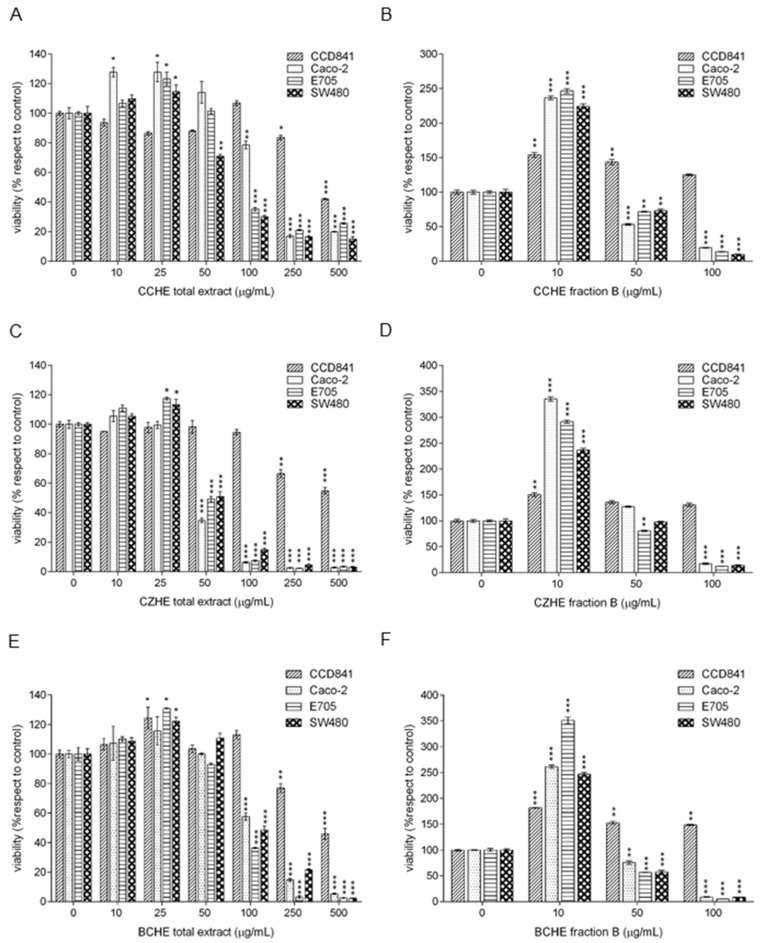
MTT viability assay on the healthy colorectal mucosa CCD841 cell line and colorectal cancer cell lines Caco-2, E705, and SW480. The cells were treated for 48 h with hydroalcoholic total extracts of the bark from *Cinnamomum cassia* (CCHE) (panel **A**) or *Cinnamomum zeylanicum* (CZHE) (panel **C**) or the buds from *Cinnamomum cassia* (BCHE) (panel **E**) at a concentration between 0 and 500 μg/mL and corresponding fractions enriched in polyphenols (panel **B**) at concentrations between 0 and 100 μg/mL (panel **B**,**D**,**F**). Statistical significance: * *p* < 0.05, ** *p* < 0.01, *** *p* < 0.001 (Dunnett’s Test).

MTT assays (Figure 2) revealed opposite biological effects of total extracts and fractions enriched in polyphenols at different doses. In particular, we observed a beneficial effect at low doses and a toxic effect at high doses: this phenomenon has been described using the term of hormesis [23]. The stimulatory effect was observed for CCHE total extract at 10 μg/mL on Caco-2 cells and 25 μg/mL on Caco-2, E705, and SW480 cells (Figure 2A), for CZHE total extract at 25 μg/mL on E705 and SW480 cells (Figure 2C), and for BCHE total extract at 25 μg/mL on CCD841, E705, and SW480 cells (Figure 2E). 

The CCHE total extract showed a significant dose-dependent cytotoxic effect on Caco-2 and E705 cell lines, starting from 100 μg/mL concentration, and on SW480, starting from 50 μg/mL (Figure 2A). The CZHE total extract proved to be the most effective, showing a significant cytotoxic effect on all colorectal cancer cell lines at the dose of 50 μg/mL, where the viability dropped to 35% for Caco-2, 49% for E705, and 51% for SW480 (Figure 2C). The BCHE total extract showed a significant cytotoxic effect on all colorectal cancer cell lines, starting from 100 μg/mL (Figure 2E). Only higher concentrations (250 and 500 μg/mL) of all total extracts had a toxic effect on the healthy colorectal mucosa CCD841 cell line.

The polyphenol-enriched fractions from CCHE, CZHE, and BCHE extracts showed a significant increase in cell viability at 10 μg/mL in all the cell lines (Figure 2B,D,F). By comparing the CZHE total extract and the corresponding fraction B, we observed that the fraction was less effective against cancer lines at both 50 and 100 μg/mL concentrations. Instead, as for fractions B from CCHE and BCHE, the 50 and 100 μg/mL doses discriminated between the healthy line and the three tumor lines, showing a beneficial effect on the healthy colorectal mucosa CCD841 cell line and a cytotoxic effect on Caco-2, E705, and SW480 cell lines. By comparing the effect of total extracts and the corresponding polyphenol-enriched fractions at the same concentrations, the last fractions were more effective on tumor cell lines. Moreover, fractions B from CCHE and BCHE were more cytotoxic on cancer cells than fraction B from CZHE extract.

Noteworthy, fractions B at all concentrations did not affect the healthy cell line, demonstrating a selectivity against colorectal cancer cell lines.

**Figure 3:**
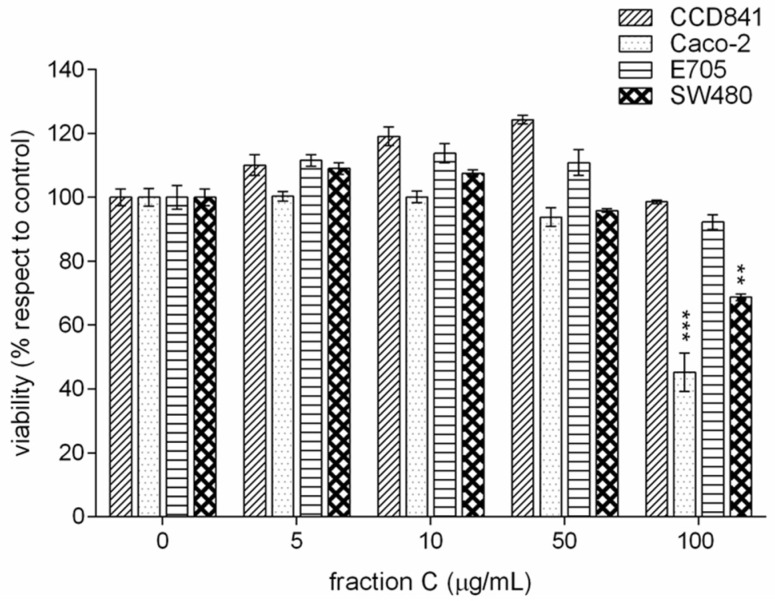
MTT viability assay on healthy colorectal mucosa CCD841 cell line and colorectal cancer cell lines Caco-2, E705, and SW480. The cells were treated for 48 h with fraction C enriched in cinnamaldehyde at concentrations between 0 and 100 μg/mL. Statistical significance: ** *p* < 0.01, *** *p* < 0.001 (Dunnett’s Test).

Fraction C enriched in cinnamaldehyde showed no significant effect on cell viability in any line up to a concentration of 50 μg/mL. We observed a toxic effect only on Caco-2 and SW480 lines at 100 μg/mL (Figure 3).

### 2.3. Additive Effect of Cinnamon Fractions Enriched in Polyphenols

**Figure 4:**
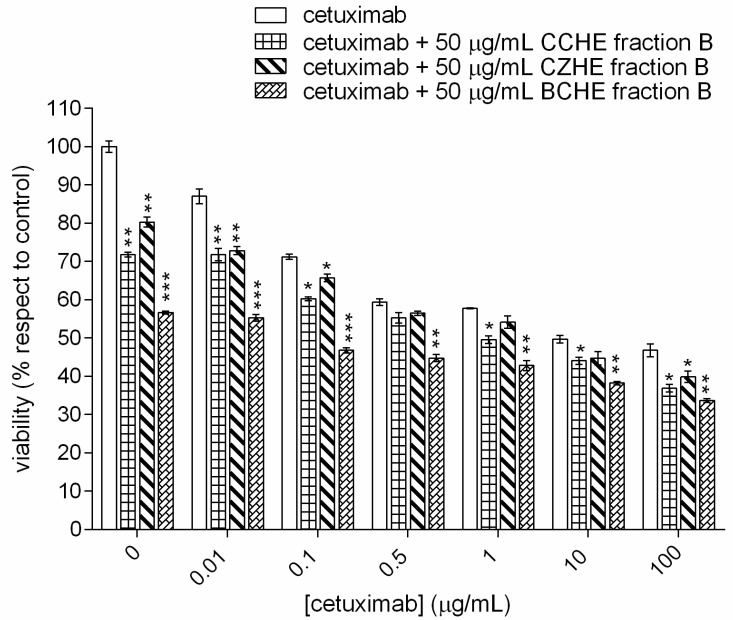
MTT viability assay on colorectal cancer E705 cell line. The cells were treated for 48 h with different concentrations of cetuximab (0–100 μg/mL) and at a fixed concentration of cinnamon fraction B (50 μg/mL). Statistical significance: * *p* < 0.05, ** *p* < 0.01, *** *p* < 0.001 (Dunnett’s Test).

To evaluate the combined effect of cinnamon fraction B and cetuximab, we used the cetuximab-sensitive E705 cell line, and the cells were treated with different concentrations of cetuximab (0–100 μg/mL) and at a fixed concentration of cinnamon fraction B (50 μg/mL). As reported in Figure 4, an additive cytotoxic effect was observed by adding a fixed dose of polyphenol-enriched fraction from each extract to different doses of cetuximab; in particular, the most significant effect was observed, for all samples, at a cetuximab concentration of 0.01 μg/mL.

Furthermore, the fraction enriched in polyphenols from cinnamon bud extract (BCHE) was more effective at all cetuximab doses than the other two fractions. This fraction at 50 μg/mL was more effective than cetuximab at 0.1 μg/mL, and the effect is the same as the one shown by the drug at concentrations between 0.5 and 1 μg/mL.

### 2.4. Fractions Enriched in Polyphenols Induce Apoptosis in Colorectal Cancer Cell Lines

**Figure 5:**
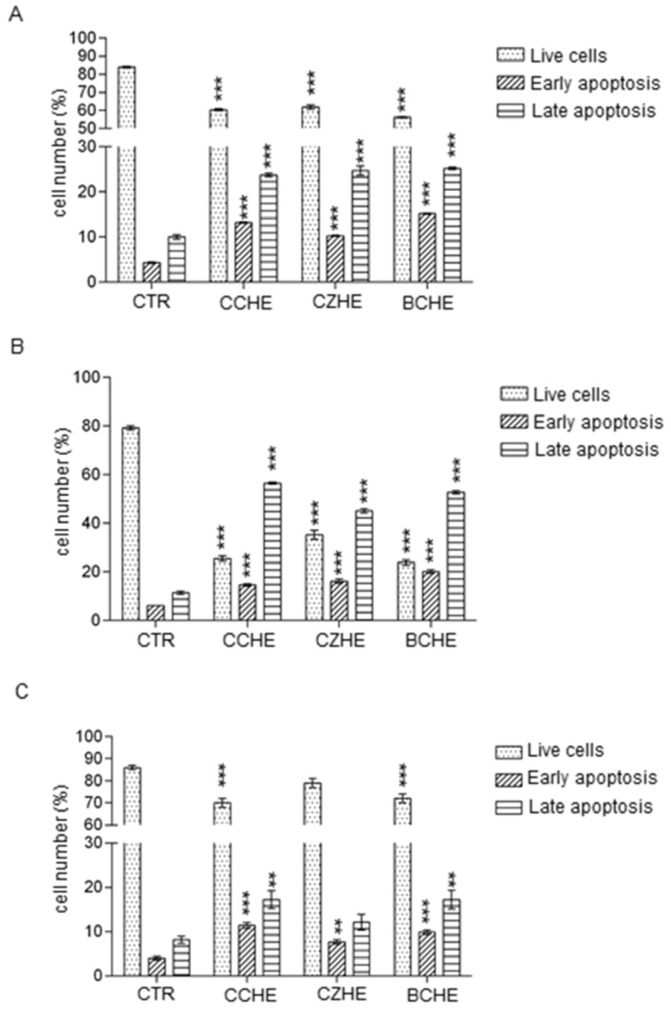
Apoptosis analysis by flow cytometry in Caco-2 (panel **A**), E705 (panel **B**), and SW480 (panel **C**) cell lines. The results are expressed as a percentage of total cell numbers and are mean ± standard error (SE) of three individual experiments. Statistically significant: ** *p* < 0.01, *** *p* < 0.001 (Dunnett’s Test).

To examine whether the decrease in cell viability was due to apoptosis, we performed a flow cytometric analysis for Annexin V-FITC and PI and found that fractions enriched in polyphenols obtained from CCHE, CZHE, and BCHE hydroalcoholic extracts at a dose of 50 μg/mL induced apoptosis in all colorectal cancer cell lines. As reported in Figure 5A and Supplementary Material—Figure S6, in Caco-2 cells, the three analyzed fractions displayed a similar effect, showing a significant decrease in live cells and a significant increase in early and late apoptotic cells.

A stronger effect of all three fractions was observed on E705 cells, where the late apoptotic cells are 56%, 44%, and 52% after treatment with CCHE fraction B, CZHE fraction B, and CCHE fraction B, respectively (Figure 5B and Supplementary Material—Figure S7). As reported in Figure 5C and Supplementary Material—Figure S8, the treatment with fractions B from all hydroalcoholic extracts was less effective in inducing apoptosis in SW480 than in other cell lines.

### 2.5. Fractions Enriched in Polyphenols Induce Apoptosis through ERK Activation and Reduce Mitochondrial Membrane Potential

**Figure 6:**
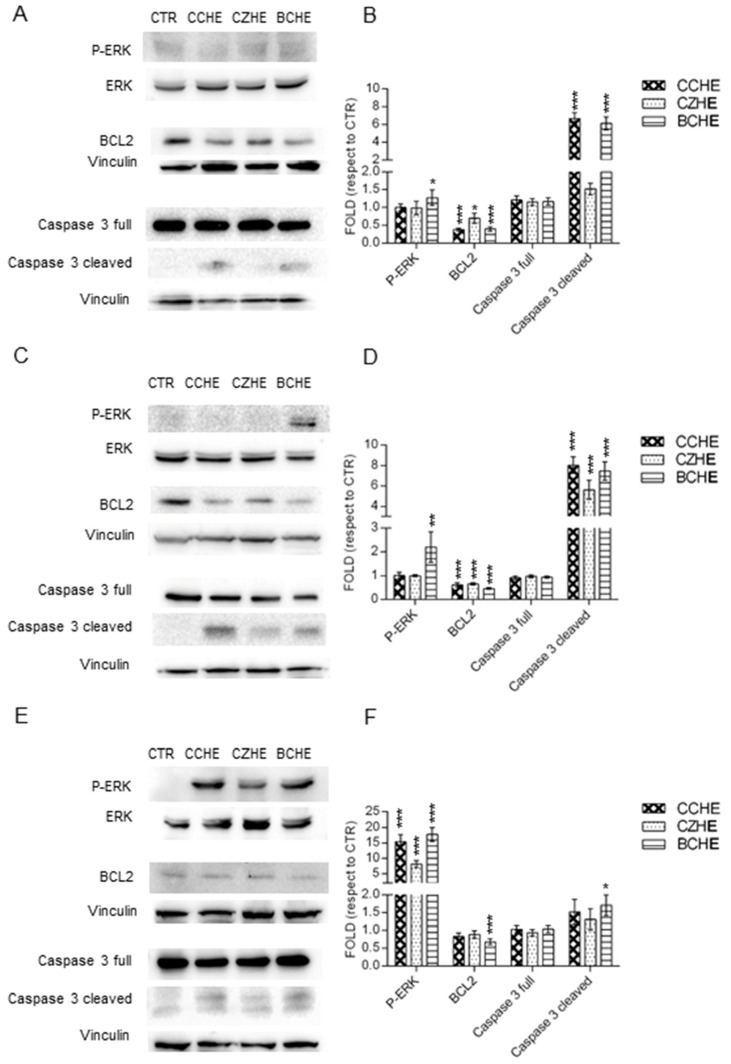
Western blot analysis. Representative Western blot analyses performed on Caco-2 (panel **A**), E705 (panel **C**), and SW480 (panel **E**) cell lines treated for 48 h with 50 μg/mL of fractions B from hydroalcoholic extracts of *Cinnamomum cassia* bark (CCHE), *Cinnamomum zeylanicum* bark (CZHE), and *Cinnamomum cassia* buds (BCHE). Protein extracts were separated via SDS-PAGE and the membranes were probed with anti-P-ERK, anti-ERK, anti-BCL2, and anti-caspase 3 antibodies. Vinculin was used as a loading control. The experiments were performed in triplicate. Densitometric analyses were performed with Scion Image Software v. 4.0 (panels **B**,**D**,**F**). Values are expressed as fold with respect to the control condition and are presented as means ± standard error (SE) of three individual experiments. * *p* < 0.05, ** *p* < 0.01, *** *p* < 0.001.

Western blot analyses showed different molecular mechanisms of apoptosis. In Caco-2 cells, only the treatment with BCHE fraction B induced a weak ERK activation. CCHE and BCHE fractions B induced a decrease in anti-apoptotic BCL2 factor and caspase 3 cleavage and activation (Figure 6A).

In E705 cells, only the treatment with BCHE fraction B induced ERK activation, but treatment with any fraction B induced a BCL2 decrease and caspase 3 activation (Figure 6B). In SW480, we observed a significant ERK activation by all fractions B, with a weak BCL2 decrease and a weak caspase 3 activation (Figure 6C).

**Figure 7:**
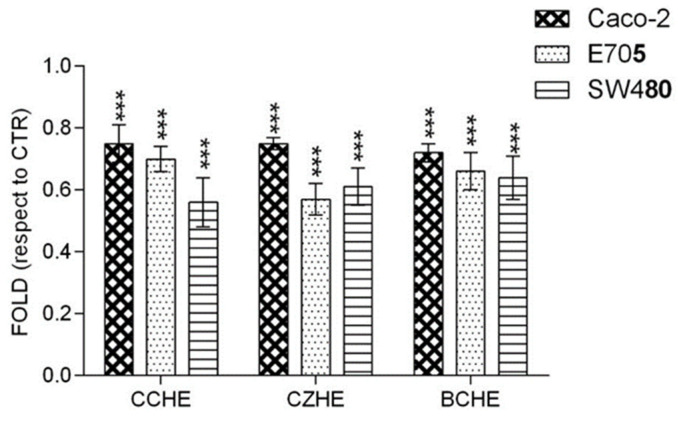
Mitochondrial membrane potential analysis. After treatment for 48 h with 50 μg/mL of fractions B from hydroalcoholic extracts of *Cinnamomum cassia* bark (CCHE), *Cinnamomum zeylanicum* bark (CZHE), and *Cinnamomum cassia* buds (BCHE), the cells were incubated with 40 nM DiOC6 and the level of fluorescence was evaluated. The results are representative of three independent experiments. Statistically significant: *** *p* < 0.001 (Dunnett’s Test).

Mitochondria membrane potential (∆ψ_m_) is an important parameter of mitochondrial function and an indicator of cell health. Treatment with CCHE, CZHE, and BCHE fractions B induced a loss of DiOC6 fluorescence in all colorectal cancer cell lines, indicating disruption of the mitochondrial inner transmembrane potential (Figure 7). 

The reduction in ∆ψ_m_ suggests the loss of mitochondrial membrane integrity, reflecting the initiation of the proapoptotic signal.

## 3. Discussion

The hydroalcoholic extracts and fractions enriched in polyphenols (B) of bark from *Cinnamomum cassia* (CCHE) or *Cinnamomum zeylanicum* (CZHE) or buds from *Cinnamomum cassia* (BCHE) induced biologically opposite effects at different doses on the viability of healthy colorectal mucosa CCD841 cell line and colorectal cancer cell lines Caco-2, E705, and SW480. The term hormesis has long been used to describe the phenomenon where a specific chemical can induce a stimulatory or beneficial effect at low doses and an inhibitory or toxic effect at high doses [24,25]. At higher doses, all extracts were found to be selectively cytotoxic toward cancer cells.

Although fractions B obtained from the three different cinnamon extracts showed different UPLC–HRMS profiles (Figure 1 and Table 1), procyanidins were found to be the predominant constituents in all cases. In fractions B from CCHE and CZHE extracts, they are mainly represented by Type A procyanidins (mostly trimers). B-type procyanidins, as well as other flavonoids in lower concentrations, have been found in fraction B from CCHE. Fraction B from BCHE is instead the richest in terms of chemical diversity. Notably, although the Type B procyanidins oligomers are the most represented species, several flavonol and phenolic derivatives emerged from UPLC–HRMS analysis, including different glycosides of quercetin and kaempferol (such as quercitrin, isoquercitrin, astragalin, avicularin, and juglalin).

Fractions B from both CCHE and BCHE extracts were found to be more cytotoxic when compared to their respective original extracts and also to the fraction B obtained from CZHE extract, suggesting a role in their antiproliferative activity either for B-type procyanidins or for flavonoid glycosides. Interestingly, fraction C, mainly containing cinnamaldehyde and related compounds, when tested at the same concentrations of fractions B, was not found to be effective in reducing cell viability in any cell line, with the only exception of the slight toxic effect on Caco-2 and SW480 lines at the maximum tested concentration. As already mentioned, the antiproliferative activity of cinnamaldehyde on some tumor cell lines was previously described as exerted through different mechanisms, including induction of apoptosis, cell cycle arrest, interruption in angiogenesis, free radical scavenging, inhibition of inflammation, and interference with cellular invasion and metastasis [21,26]. However, Franziska Roth-Walter et al. [27] reported that the treatment with cinnamaldehyde led to inhibition of cell viability and proliferation and induced apoptosis in primary and immortalized immune cells, clearly indicating that, despite its anti-carcinogenic property, cinnamaldehyde administration to cancer patients might be contraindicated due to its ability to inhibit immune cell activation. This side effect would be even more limiting considering the high concentrations required to have a significant antiproliferative activity against the CRC cell lines here tested. These observations are even more important when considering the effect of the co-administration of fractions B with the monoclonal antibody cetuximab.

When fraction B antiproliferative activity was tested on E705 cancer cells, by incubating each fraction B at 50 μg/mL concentration in the presence of different cetuximab doses, all fractions showed an additional cytotoxic effect. Interestingly, fraction B from BCHE extract was able to reduce cell viability to almost 50% in the absence of cetuximab and was found to have the same antiproliferative effect at doses of cetuximab between 1 and 10 μg/mL. This finding suggests a potentially very interesting use of cinnamon extracts enriched in polyphenols in chemotherapy, allowing one to reduce cetuximab doses, leading to a decrease in its toxic side effects as well [28]. Notably, procyanidins can also exert an immunosuppressive effect, mainly attributed to the inhibition of T cell functions by A-type procyanidins [29]. However, according to our data, fraction B from BCHE extract contains only B-type procyanidins, thus making it the best candidate for the preparation of nutraceuticals to be co-administered with cetuximab.

To understand the molecular mechanisms involved in cinnamon polyphenol cytotoxicity towards cancer cells, apoptosis was assessed. All cinnamon fractions B up-regulated the expression levels of cleaved caspase 3 while down-regulating those of Bcl-2. Moreover, they all induced apoptosis increasing ERK phosphorylation in colorectal cancer cell lines.

Contrary to the well-established role of MAPK signaling in promoting cell proliferation and survival, growing evidence suggests that the ERK signaling can mediate proapoptotic signaling. An increasing number of compounds, including betulinic acid, quercetin, kaempferol, and piperlongumine, have been reported to exert apoptosis-inducing effects through ERK activation [30]. Intriguingly, both quercetin and kaempferol glycosides were found in extracts of buds from *Cinnamomum cassia* (BCHE).

Finally, the decrease in mitochondrial membrane potential, induced by the three cinnamon fractions B is well in accordance with data reported in the literature, showing that cisplatin-induced apoptosis requires ERK activation to induce mitochondrial membrane depolarization and cytochrome *c* release, as well as caspase 3 activation [31]. Moreover, Koppikar et al. demonstrated that cinnamon extracts exhibited a potent antineoplastic effect on cervical cancer cells through loss of mitochondrial membrane potential, leading to apoptosis [16]. Limitations of our study are represented by the reduced number of cell lines analyzed and the absence of molecular and biochemical analyses in experiments using animals, which will be the object of future studies. Nevertheless, this preliminary study has very interesting potential clinical applications for patients treated with EGFR-targeted therapies, who could benefit from reduced cetuximab doses, and also for patients carrying KRAS mutations, who cannot be treated with these therapies due to primary resistance.

## 4. Materials and Methods

### 4.1. Cinnamon Extracts and Polyphenols-Enriched Fractions Preparation

Cinnamon bark and bud extracts and polyphenol-enriched fractions were prepared as previously described by our group [17]. Briefly, cinnamon samples were finely ground, sieved at 400 µm, and extracted using a mixture of ethanol (30%) and acidified water at pH 4.5 (70%) in an ultrasound bath at 45 °C for 60 min. Then, the solution was collected by centrifugation, filtered, concentrated under reduced pressure, and dried by lyophilization, obtaining freeze-dried cinnamon extract. Polyphenol-enriched fractions (namely, fractions B) were isolated from each cinnamon extract by preparative reversed-phase flash chromatography using a Biotage^®^ Isolera™ Prime system (Biotage AB, Uppsala, Sweden). Separations were carried out on the SNAP KP-C18 column using water (A) and methanol (B) as solvents and applying a linear gradient elution (2% B–100% B in 15 CV). Eluate subfractions were pooled in homogeneous groups and solvents were removed under reduced pressure; finally, the residues were dried by lyophilization, obtaining freeze-dried cinnamon polyphenol-enriched fractions.

### 4.2. UPLC–HRMS Characterization

High-Resolution Mass Spectrometry (HRMS) analysis of cinnamon polyphenol-enriched fractions (fraction B) was performed using the ACQUITY UPLC H-class system coupled with the Xevo G2-XS QToF Mass Spectrometer (Waters Corp., Milford, MA, USA) through an ESI source. Samples were dissolved in 90% water–10% acetonitrile at 1 mg/mL and were separated on the ACQUITY Premier HSS T3 Column (100 mm × 2.1 mm, 1.8 µm) coupled with VanGuard™ HSS T3 guard column (Waters Corp., Milford, MA, USA). The mobile phases were MS-grade water (A) and acetonitrile (B), both containing 0.1% formic acid, and analyte elution was performed according to the following gradient: 0–1 min, 5% B; 1–11 min, 5–50% B linear gradient; 11–12 min, 50–90% B, 12–15 min, isocratic 90% B, and then were equilibrated further for 4 min at the initial conditions (5% B) before the next sample injection. Elution was performed at a flow rate of 0.4 mL/min, and the injection volume was 2 μL. The column temperature was set at 40 °C. Accurate mass data were collected under negative ionization through a data-dependent acquisition mode (FastDDA) in which a full scan survey triggered the MS/MS acquisition of the five most intense ions (Top 5) in the range of 50–1200 m/z. Full scan spectra were acquired at a scan time of 0.2 s and MS/MS spectra were acquired at a scan time of 0.1 s. Dynamic collision energy was set to 6–9 V for 50 Da and 60–80 V for 1200 Da. The source parameters were as follows: electrospray capillary voltage −2 kV, source temperature 120 °C, and desolvation temperature 350 °C. The cone and desolvation gas flows were 50 and 1000 L/h, respectively. The mass spectrometer was calibrated with 0.5 M sodium formate and leucine–enkephalin (100 pg/μL) infused at 10 μL/min and was acquired every 30 s using LockMass. The MassLynx software (Waters Corp., Milford, MA, USA, version 4.2) was used for instrument control, data acquisition, and data processing. MS Dial software version 4.9.221218 (http://prime.psc.riken.jp/compms/index.html) was used for the peak picking, deconvolution and noise level setting, and identification of metabolites was performed according to their calculated accurate mass and isotopic pattern. Structures were confirmed by comparison MS/MS spectra using the metabolomics MSP spectral kit, public databases, and the literature [32,33].

### 4.3. Cell Cultures

CCD841 (ATCC^®^ CRL-1790™) human healthy mucosa cell line and CaCo-2 (ATCC^®^ HTB-37™) human colorectal cancer cell lines were grown in EMEM medium supplemented with heat-inactivated 10% fetal bovine serum (FBS), 2 mM L-glutamine, 0.1 mM non-essential amino acids, 100 U/mL penicillin, and 100 μg/mL streptomycin. E705 (kindly provided by Fondazione IRCCS Istituto Nazionale dei Tumori, Milan, Italy) and SW480 (ATCC^®^ CCL-228™) human colorectal cancer cell lines were grown in RPMI 1640 medium supplemented with heat-inactivated 10% FBS, 2 mM L-glutamine, 100 U/mL penicillin, and 100 μg/mL streptomycin. All cell lines were maintained at 37 °C in a humidified 5% CO_2_ incubator. ATCC cell lines were validated by short tandem repeat profiles that were generated by simultaneous amplification of multiple short tandem repeat loci and amelogenin (for gender identification). All the reagents for cell cultures were supplied by EuroClone (EuroClone S.p.A, Milan, Italy).

### 4.4. Viability Assay

Cell viability assay was investigated using an MTT-based in vitro toxicology assay kit (Merck KGaA, Darmstadt, Germany), according to the manufacturer’s protocols. The different cell lines were seeded in 96-well microtiter plates at a density of 1 × 10^4^ cells/well, cultured in complete medium, and, after 24 h, treated with hydroalcoholic extracts of bark from *Cinnamomum cassia* (CCHE) or *Cinnamomum zeylanicum* (CZHE) or buds from *Cinnamomum cassia* (BCHE) at a concentration between 0 and 500 μg/mL and with fractions enriched in polyphenols (B) or cinnamaldehyde (C) at concentrations between 0 and 100 μg/mL. All samples were solubilized in 10% dimethyl sulfoxide (DMSO). The DMSO concentration in the wells was 0.25% for both treated and control cells. Furthermore, 24 h after the seeding, E705 cells were treated with different concentrations of cetuximab (mAbs against EGFR; 0–100 μg/mL), at a fixed concentration of cinnamon fraction B (50 μg/mL). After 48 h at 37 °C, the medium was replaced with a complete medium without phenol red, containing 10 μL of 5 mg/mL MTT (3-(4,5-Dimethylthiazol-2-yl)-2,5-Diphenyltetrazolium Bromide). After 4 h incubation for CCD841 and 2 h for CRC cell lines, formazan crystals were solubilized with 10% Triton X-100 and 0.1 N HCl in isopropanol, and absorbance was measured at 570 nm using VICTOR^®^ Multilabel Plate Reader (PerkinElmer, Waltham, MA, USA). Cell viability was expressed as a percentage against untreated cell lines used as controls. 

### 4.5. Annexin V-FITC Assay for Apoptosis

The cells were seeded into 24-well plates at a density of 8 × 10^4^ cells/well. After 24 h of incubation, the cells were treated for 48 h with cinnamon fraction B at 50 μg/mL obtained from hydroalcoholic extracts of the bark from *Cinnamomum cassia* (CCHE) or *Cinnamomum zeylanicum* (CZHE) or the buds from *Cinnamomum cassia* (BCHE). After treatment, cells were harvested by trypsinization and stained with Annexin V/FITC and propidium iodide (PI) in a binding buffer, according to the manufacturer’s protocol (Cat n° V13242, Thermo Fisher Scientific, Waltham, MA, USA). Cells were analyzed using a flow cytometer, and flow cytometric data were analyzed using CytExpert 2.3 Software (Beckman Coulter Inc., Brea, CA, USA).

### 4.6. SDS-PAGE and Western Blotting

For Western blot analysis, the cells were seeded at 1 × 10^6^ cells/100 mm dish, and 24 h after seeding, were treated with cinnamon fraction B at 50 μg/mL obtained from hydroalcoholic extracts of the bark from *Cinnamomum cassia* (CCHE) or *Cinnamomum zeylanicum* (CZHE) or the buds from *Cinnamomum cassia* (BCHE) for 48 h. After treatment, the cells were rinsed with ice-cold PBS and lysed in RIPA buffer (50 mM Tris–HCl pH 7.5, 150 mM NaCl, 1% NP-40, 0.5% sodium deoxycholate, 0.1% SDS) containing 1 μM leupeptin, 2 μg/mL aprotinin, 1 μg/mL pepstatin, 1mM PMSF, and a phosphatase inhibitor cocktail (Merck KGaA, Darmstadt, Germany). After lysis on ice, homogenates were obtained by passing 5 times through a blunt 20-gauge needle fitted to a syringe and then centrifuged at 15,000× *g* for 30 min. Supernatants were analyzed for protein content by the BCA protein assay [34]. SDS-PAGE and Western blotting were carried out by standard procedures [35]. Sixty micrograms of proteins were separated on a 10% or 12% acrylamide/bis-acrylamide SDS-PAGE and transferred onto a nitrocellulose membrane (Millipore, Billerica, MA, USA). The membrane was subsequently blocked for 30 min in 5% (*w/v*) dried milk in PBS, probed overnight at 4 °C with the appropriate primary antibodies, and visualized using the ECL detection system (EuroClone S.p.A, Milan, Italy). Protein levels were quantified by densitometry of immunoblots using Scion Image software v. 4.0 (Scion Corp., Frederick, MD, USA). The following primary antibodies were used: anti-P-ERK (dilution 1:1000), anti-ERK (dilution 1:1000), anti-BCL2 (dilution 1:1000), anti-caspase 3 (dilution 1:1000) (purchased by Cell Signaling Technology, Danvers, MA, USA), and anti-vinculin (dilution 1:10,000) (purchased from Merck KGaA, Darmstadt, Germany). IgG HRP anti-rabbit and anti-mouse conjugated secondary antibodies (purchased by Cell Signaling Technology, Danvers, MA, USA) were diluted 1:5000.

### 4.7. Mitochondrial Transmembrane Potential (MTP) Assay

MTP alterations were assayed through fluorescence analysis, using the green fluorescent membrane dye 3,3′-dihexyloxacarbocyanine iodide (DiOC6), which accumulates in mitochondria due to their negative membrane potential and can be applied to monitor the mitochondrial membrane potential. The different cell lines were seeded in 96-well microtiter plates at a density of 1 × 10^4^ cells/well, cultured in complete medium, and, after 24 h, treated with cinnamon fraction B at 50 μg/mL, obtained from hydroalcoholic extracts of bark from *Cinnamomum cassia* (CCHE) or *Cinnamomum zeylanicum* (CZHE) or buds from *Cinnamomum cassia* (BCHE) for 48 h. After treatment, cells were incubated with 40 nM DiOC6 (diluted in PBS) for 20 min at 37 °C in the dark and rinsed with PBS; fluorescence was measured, following PBS addition (excitation = 484 nm; emission = 501 nm), using VICTOR^®^ Multilabel plate reader (PerkinElmer, Waltham, MA, USA).

### 4.8. Statistical Analysis

All the experiments were carried out in triplicate. The samples were compared to their reference controls and the data were tested by Dunnett’s multiple comparison procedure (GraphPad Prism Software v. 6.01). Results were considered statistically significant at *p* < 0.05.

## 5. Conclusions

Collectively, our findings imply that cinnamon polyphenol-enriched fraction has great potential in being utilized for the prevention and treatment of CRC. Cinnamon buds, whose polyphenolic fraction differs significantly from that of the barks, are the matrix with the best biological activity. This is also very interesting since, among the different parts of the cinnamon plant, the buds, to date, are by far the least studied.

Moreover, the knowledge of the specific effects of diet components can be useful in the prevention and treatment of several diseases, including gastrointestinal disease and cancer. In this scenario, this work provides the rationale for the use of specific dietary components for prevention and personalized adjuvant therapies.

## Figures and Tables

**Table 1:** Detailed UPLC–HRMS identification of the main components in polyphenol-enriched fractions B obtained from *Cinnamomum cassia* buds (BC) and bark (CC) and *Cinnamomum zeylanicum* bark (CZ) extracts.

#	RT (Min)	ID	Molecular Formula	Monoisotopic Mass	Experimental m/z	Adduct Type	Abs. Error (ppm)	Source
1	3.85	Cinnacassoside C	C_19_H_28_O_13_	464.1530	463.1458	[M-H]^−^	0.21	CZ
2	4.20	A-type ProCy tetramer	C_60_H_48_O_24_	1152.2536	1151.2458	[M-H]^−^	0.37	CZ
3	4.25	B-type ProCy dimer	C_30_H_26_O_12_	578.1424	577.1354	[M-H]^−^	0.50	CC
4	4.27	A-type ProCy tetramer	C_60_H_48_O_24_	1152.2540	1151.245	[M-H]^−^	1.11	CC
5	4.41	Phenolic glycoside (NCGC00180160-01)	C_19_H_28_O_12_	448.1581	493.1564	[M+FA-H]^−^	0.31	CC
6	4.49	B-type ProCy dimer	C_30_H_26_O_12_	578.1424	577.1348	[M-H]^−^	0.56	CC
7	4.65	A-type ProCy pentamer	C_75_H_62_O_30_	1440.3169	719.1531	[M-2H]^2−^	2.7	CZ
8	4.67	Epicatechin	C_15_H_14_O_6_	290.0790	289.0716	[M-H]^−^	0.47	CC
11	4.83	B-type ProCy trimer	C_45_H_38_O_18_	866.2058	865.1968	[M-H]^−^	1.79	BC, CC
12	4.85	A-type ProCy pentamer	C_75_H_62_O_30_	1440.3169	719.1543	[M-2H]^−^	4.31	CZ
13	4.88	3,4,5-Trimethoxyphenyl 6-O-apiofuranosylglucopyranoside	C_20_H_30_O_13_	478.1686	477.1619	[M-H]^−^	1.05	CZ
14	4.89	A-type ProCy pentamer	C_75_H_60_O_30_	1440.3170	719.1532	[M-2H]^2−^	2.79	CC
15	4.92	B-type ProCy dimer	C_30_H_26_O_12_	578.1424	577.1345	[M-H]^−^	1.09	BC
16	4.98	A-type ProCy tetramer	C_60_H_48_O_24_	1152.2540	1151.246	[M-H]^−^	0.26	CC
17	5.01	B-type ProCy dimer	C_30_H_26_O_12_	578.1424	577.1352	[M-H]^−^	0.09	BC, CC, CZ
18	5.16	B-type ProCy tetramer	C_60_H_50_O_24_	1154.2690	1153.2598	[M-H]^−^	1.87	BC
19	5.18	Benzyl β-primeveroside	C_18_H_26_O_10_	402.1526	447.1505	[M+FA-H]^−^	0.54	CZ, CC
20	5.24	A-type ProCy pentamer	C_75_H_60_O_30_	1440.3170	719.1524	[M-2H]^2−^	1.68	CC
21	5.33	A-type ProCy trimer	C_45_H_36_O_18_	864.1902	863.1835	[M-H]^−^	0.75	CC
22	5.37	Phenolic glycosides	C_18_H_24_O_11_	416.1319	415.1244	[M-H]^−^	0.47	CZ
23	5.37	Catechin	C_15_H_14_O_6_	290.0790	289.0718	[M-H]^−^	0.05	CC, BC
24	5.46	A-type ProCy tetramer	C_60_H_48_O_24_	1152.254	1151.245	[M-H]^−^	1.11	CC
25	5.48	A-type ProCy trimer	C_45_H_36_O_18_	864.1902	863.1821	[M-H]^−^	0.95	CZ
26	5.53	B-type ProCy trimer	C_45_H_38_O_18_	866.2058	865.1997	[M-H]^−^	1.35	BC
27	5.54	A-type ProCy trimer	C_45_H_36_O_18_	864.1902	863.1946	[M-H]^−^	2.02	CC
28	5.63	A-type ProCy tetramer	C_60_H_48_O_24_	1152.2536	1151.2454	[M-H]^−^	0.79	CZ
29	5.68	B-type ProCy tetramer	C_60_H_50_O_24_	1154.2690	1153.264	[M-H]^−^	1.83	BC
30	5.68	A-type ProCy tetramer	C_60_H_48_O_24_	1152.2540	1151.2469	[M-H]^−^	0.95	CC
31	5.73	B-type ProCy pentamer	C_75_H_62_O_30_	1442.3330	720.1598	[M-2H]^2−^	0.69	BC
32	5.77	B-type ProCy dimer	C_30_H_26_O_12_	578.1424	577.1357	[M-H]^−^	1.02	CC
33	5.78	B-type ProCy trimer	C_45_H_38_O_18_	866.2058	865.1985	[M-H]^−^	0.01	BC
34	5.83	B-type ProCy pentamer	C_75_H_62_O_30_	1442.3330	720.1614	[M-2H]^2−^	2.89	BC
35	5.88	A-type ProCy trimer	C_45_H_36_O_18_	864.1902	863.1832	[M-H]^−^	0.39	CC
36	5.90	B-type ProCy hexamer	C_90_H_75_O_36_	1731.4040	864.1923	[M-2H]^2−^	1.54	BC
37	5.96	B-type ProCy hexamer	C_90_H_75_O_36_	1731.4040	864.1924	[M-2H]^2−^	1.68	BC
38	6.04	B-type ProCy heptamer	C_105_H_86_O_42_	2018.4590	1008.2208	[M-2H]^2−^	1.9	BC
39	6.08	A-type ProCy trimer	C_45_H_36_O_18_	864.1902	863.1827	[M-H]^−^	0.17	CC, CZ
40	6.10	Phenylethyl primeveroside	C_19_H_28_O_10_	416.1682	461.1678	[M+FA-H]^−^	1.12	BC, CC
41	6.15	Quercetin 3-vicianoside	C_26_H_28_O_16_	596.1377	595.1301	[M-H]^−^	0.66	BC
42	6.16	4-Hydroxyacetophenone 4-O-(6′-O-beta-D-apiofuranosyl)-beta-D-glucopyranoside	C_19_H_26_O_11_	430.1475	429.1399	[M-H]^−^	0.73	CZ
43	6.29	Lignan glycoside	C_32_H_44_O_17_	700.2579	699.2487	[M-H]^−^	2.74	CZ
44	6.36	Lusitanicoside	C_21_H_30_O_10_	442.1839	441.1765	[M-H]^−^	0.24	CZ
45	6.37	B-type ProCy trimer	C_45_H_38_O_18_	866.2058	865.1979	[M-H]^−^	0.79	BC
46	6.44	B-type ProCy dimer	C_30_H_26_O_12_	578.1424	577.1352	[M-H]^−^	0.07	BC, CC
47	6.50	Isoquercitrin	C_21_H_20_O_12_	464.0955	463.0887	[M-H]^−^	0.98	BC
48	6.54	Cichorioside L	C_25_H_38_O_11_	514.2414	559.2401	[M+FA-H]^−^	1.55	BC
49	6.58	Ptelatoside B	C_20_H_28_O_10_	428.1682	473.1667	[M+FA-H]^−^	0.63	BC, CC, CZ
50	6.61	A-type ProCy trimer	C_45_H_34_O_18_	862.1745	861.1676	[M-H]^−^	0.46	CC
51	6.66	Quercetin 3-xylosyl-(1-2)-alpha-L-arabinofuranoside	C_25_H_26_O_15_	566.1272	565.1201	[M-H]^−^	0.29	BC
52	6.69	Phenolic glycosides	C_20_H_28_O_10_	428.1683	427.1608	[M-H]^−^	0.41	CZ
53	6.72	A-type ProCy dimer	C_30_H_24_O_12_	576.1268	575.1193	[M-H]^−^	0.31	CC
54	6.76	Phenolic glycoside	C_20_H_28_O_10_	428.1682	473.1666	[M+FA-H]^−^	0.37	CC
55	6.76	Flavonoid glycoside	C_39_H_34_O_13_	710.1999	709.1921	[M-H]^−^	0.74	BC
56	6.82	Rosavin	C_20_H_28_O_10_	428.1682	427.1614	[M-H]^−^	1.02	BC
57	6.92	Phenethyl rutinoside	C_20_H_30_O_10_	430.1839	475.1819	[M+FA-H]^−^	1.45	CC
58	6.92	Avicularin	C_20_H_18_O_11_	434.0849	433.0781	[M-H]^−^	0.99	BC
59	6.93	Astragalin	C_21_H_20_O_11_	448.1006	447.0932	[M-H]^−^	0.19	BC
60	7.02	Poncirin chalcone	C_28_H_34_O_14_	594.1949	593.1870	[M-H]^−^	1.06	CC
61	7.03	Quercetin 3-(2-xylosylrhamnoside)	C_26_H_28_O_15_	580.1428	579.1353	[M-H]^−^	0.5	BC
62	7.11	A-type procyanidin trimer	C_45_H_34_O_18_	862.1745	861.1685	[M-H]^−^	1.41	CZ
63	7.11	Leeaoside	C_24_H_40_O_11_	504.2571	549.2559	[M+FA-H]^−^	1.19	CZ
64	7.18	Quercitrin	C_21_H_20_O_11_	448.1006	447.0929	[M-H]^−^	0.87	BC
65	7.33	Juglalin	C_20_H_18_O_10_	418.0900	417.0820	[M-H]^−^	1.72	BC
66	7.45	Phenolic glycoside	C_20_H_28_O_10_	428.1682	427.1605	[M-H]^−^	1.19	BC
67	7.59	Kaempferol-O-glycoside	C_26_H_28_O_14_	564.1479	563.1399	[M-H]^−^	1.31	BC
68	7.70	Kaempferin	C_21_H_20_O_10_	431.0983	431.0983	[M-H]^−^	0.07	BC
69	7.74	Flavonoid glycoside	C_39_H_34_O_13_	710.1999	709.1918	[M-H]^−^	1.17	BC
70	7.85	Secoisolariciresinol	C_20_H_26_O_6_	362.1729	407.1704	[M+FA-H]^−^	1.87	BC, CZ
71	8.04	Flavanone glycoside	C_24_H_22_O_7_	422.1366	421.1289	[M-H]^−^	0.88	BC
72	8.09	Cinnammic acid	C_9_H_8_O_2_	148.0524	147.0448	[M-H]^−^	2.28	BC
73	8.55	Piperic acid	C_12_H_10_O_4_	218.0579	217.0507	[M-H]^−^	0.33	CZ

## Data Availability

Data are contained within the article and Supplementary Materials.

## Supplementary Materials

The supporting information can be downloaded at: https://www.mdpi.com/article/10.3390/ijms242216117/s1.
