# Supplementary material for: Adjuvant Effect of Cinnamon Polyphenolic Components in Colorectal Cancer Cell Lines
Source: Int J Mol Sci. 2023 Nov 9;24(22):16117. doi: 10.3390/ijms242216117 (PMC10670979; doi:10.3390/ijms242216117)
Supplement: Supplementary file 1 [file ijms-24-16117-s001.zip › ijms-2670928-supplementary.pdf]

Adjuvant effect of cinnamon polyphenolic components in colorectal cancer cell lines

Alessandro Palmioli, Matilde Forcella, Monica Oldani, Irene Angotti, Grazia Sacco, Paola Fusi, Cristina Airoldi*

Department of Biotechnology and Biosciences, University of Milano-Bicocca, P.zza della Scienza, 2, 20126, Milan, Italy.

Supplementary Materials

Table of contents

Figure S1. NMR metabolic profiling of cinnamon extracts

Figure S2. Fractionation of cinnamon extract CCHE and NMR spectra of fractions B and C.

Figure S3. Chromatographic profile of the fractionation of BCHE extract obtained by preparative reverse phase C18 chromatography.

Figure S4. Chromatographic profile of the fractionation of CZHE extract obtained by preparative reverse phase C18 chromatography.

Figure S5. MS/MS spectra of the major compounds identified in polyphenols-enriched fraction (fraction B) obtained from BCHE extract.

Figure S6. Apoptosis analysis by flow cytometry in Caco-2 cell line – scatter plots.

Figure S7. Apoptosis analysis by flow cytometry in E705 cell line – scatter plots.

Figure S8. Apoptosis analysis by flow cytometry in SW480 cell line – scatter plots.

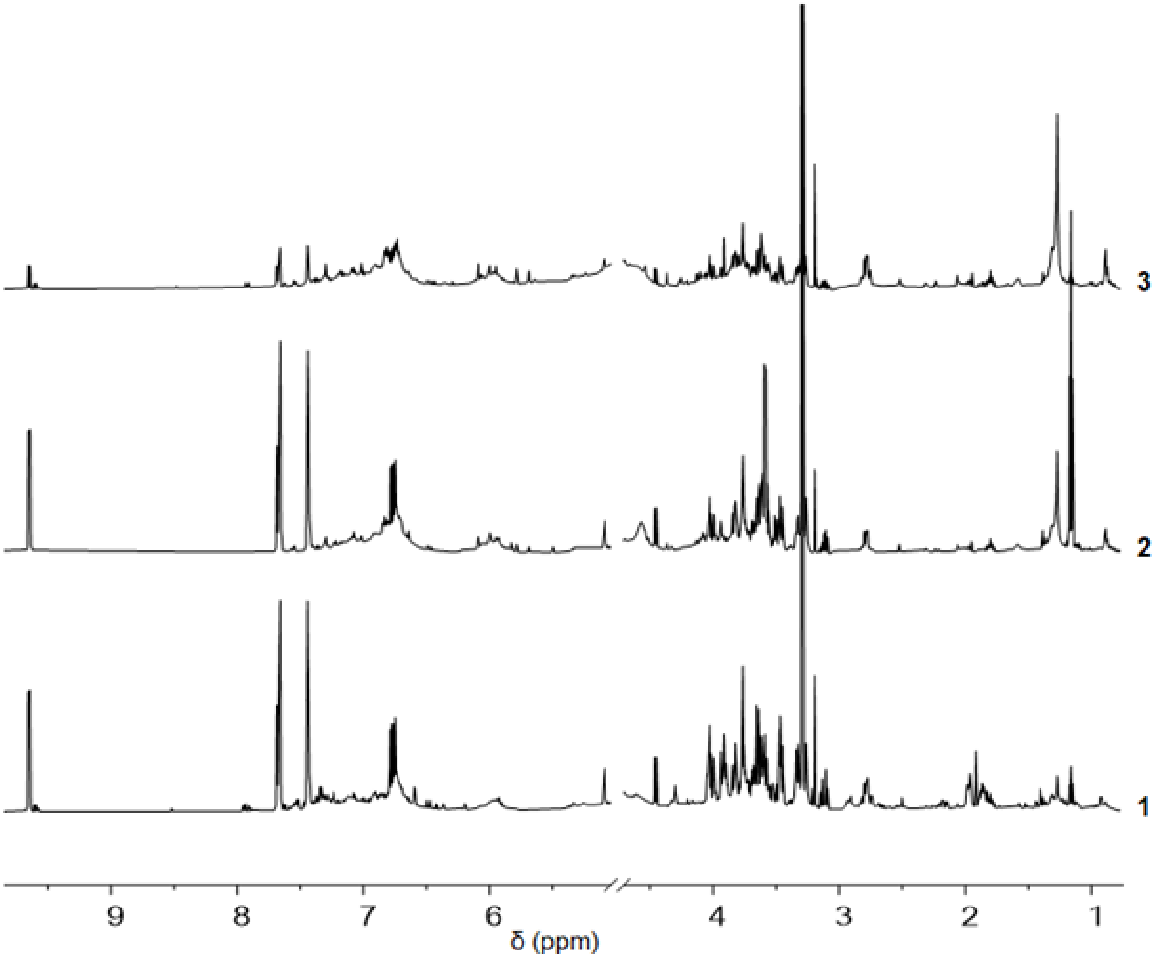

Figure S1. NMR metabolic profiling of cinnamon extracts. ^{1}H-NMR spectra of extracts of cinnamon buds (**1**) and bark (**2**, *C. cassia*; **3**, *C zeylanicum*) obtained after hydroalcoholic extraction (water:ethanol 7:3). ^{1}H-NMR spectra were recorded on 15 mg/mL samples dissolved in CD_3OD with 1 mM DSS, with *noesygppr1d* acquisition pulse sequence, at 600 MHz, 25 °C. From ref.[16]

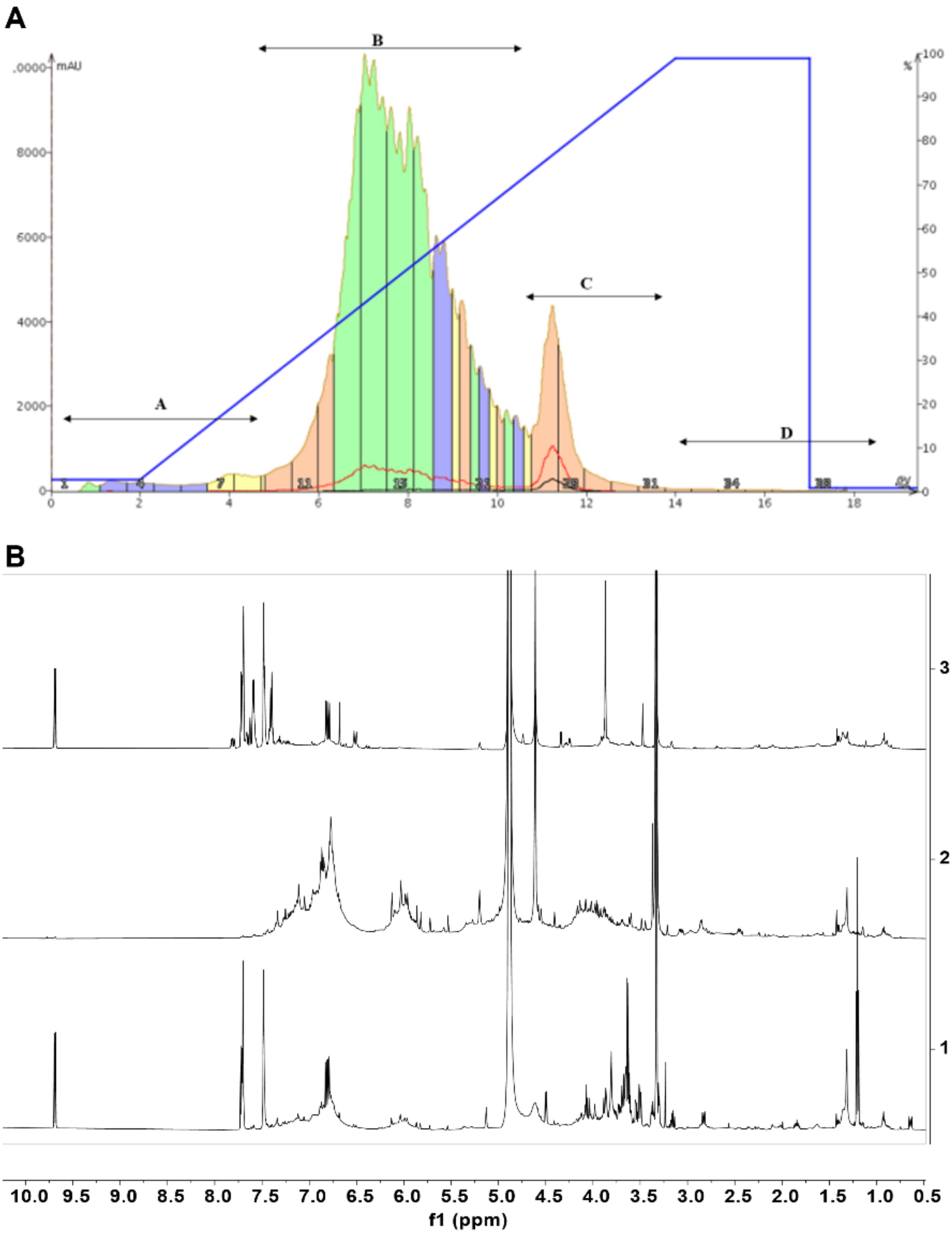

Figure S2. Fractionation of cinnamon extract CCHE and NMR spectra of fractions B and C. (**A**) Chromatographic profile of the fractionation of CCHE extract (as representative example) obtained by preparative reverse phase C18 chromatography (linear elution gradient from 2% to 100% MeOH in 15 CV). (**B**) ^{1}H-NMR spectra of the chromatographic fractions **B** (**2**) and **C** (**3**) in comparison to the ^{1}H-NMR profile of the total extract (**1**). ^{1}H-NMR spectra were recorded on 5 mg/mL samples dissolved in CD_3OD, 25 °C, at 600 MHz. From ref.[16]

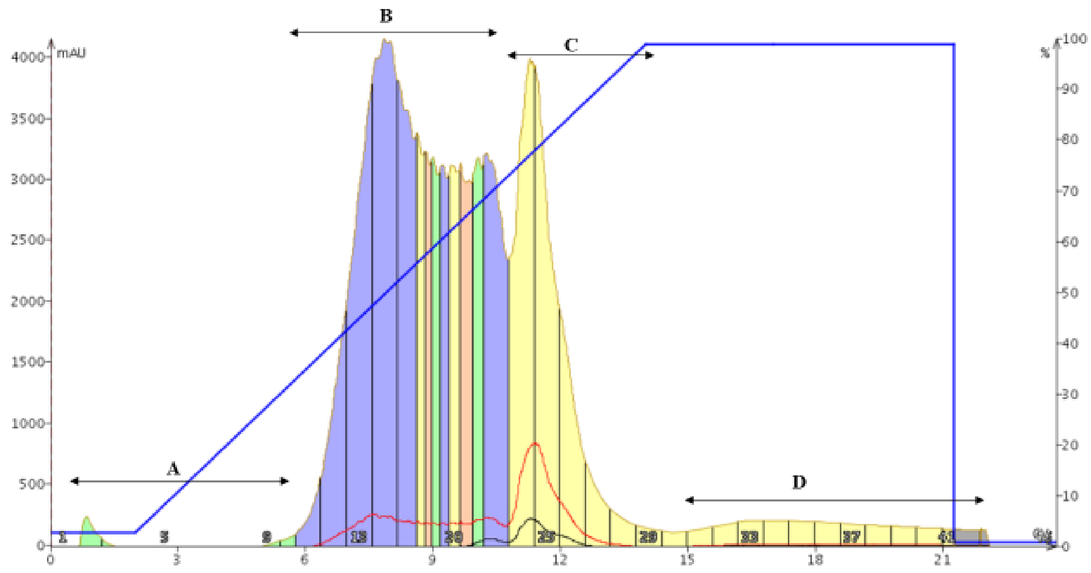

Figure S3. Chromatographic profile of the fractionation of BCHE extract obtained by preparative reverse phase C18 chromatography (linear elution gradient from 2% to 100% MeOH in 15 CV).

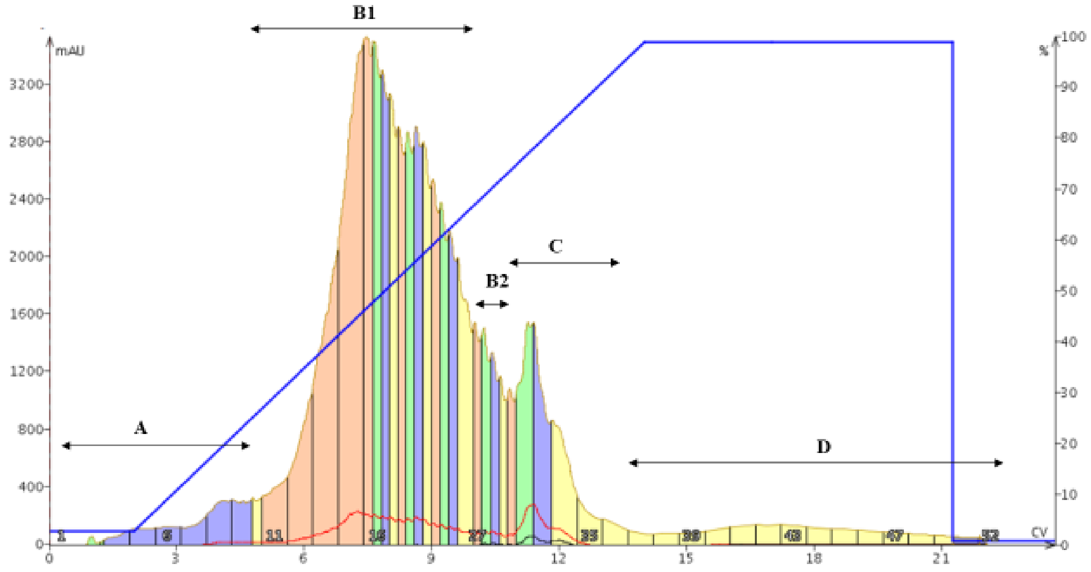

Figure S4. Chromatographic profile of the fractionation of CZHE extract obtained by preparative reverse phase C18 chromatography (linear elution gradient from 2% to 100% MeOH in 15 CV).

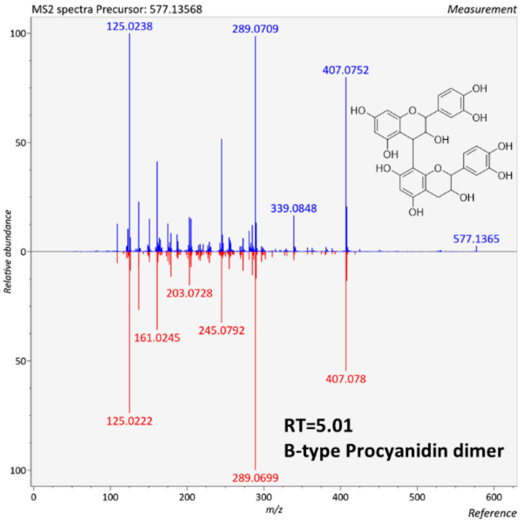
MS2 spectra Precursor: 577.13568
Measurement
125.0238
289.0709
407.0752
339.0848
577.1365
203.0728
161.0245
245.0792
407.078
125.0222
289.0699
RT=5.01
B-type Procyanidin dimer
Relative abundance
m/z
Reference

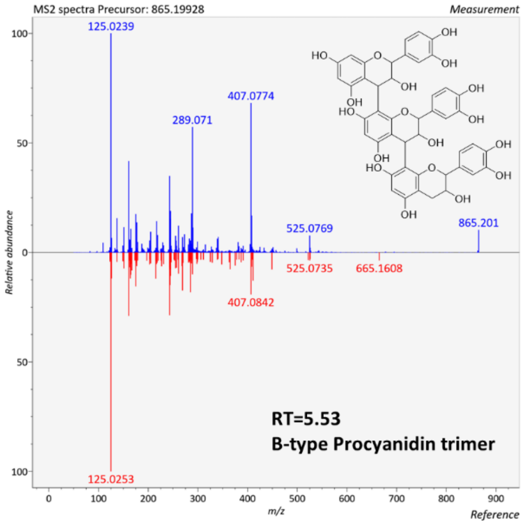
MS2 spectra Precursor: 865.19928
Measurement
125.0239
407.0774
289.071
525.0769
865.201
525.0735
665.1608
407.0842
125.0253
RT=5.53
B-type Procyanidin trimer
Relative abundance
m/z
Reference

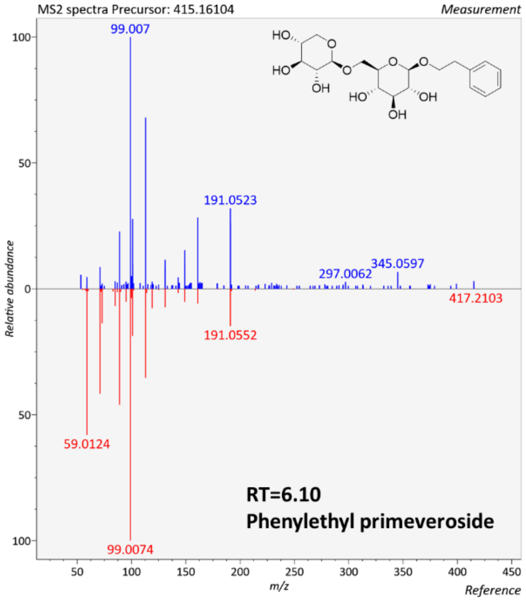
MS2 spectra Precursor: 415.16104
Measurement
99.007
191.0523
297.0062
345.0597
417.2103
191.0552
59.0124
99.0074
RT=6.10
Phenylethyl primeveroside
Relative abundance
m/z
Reference

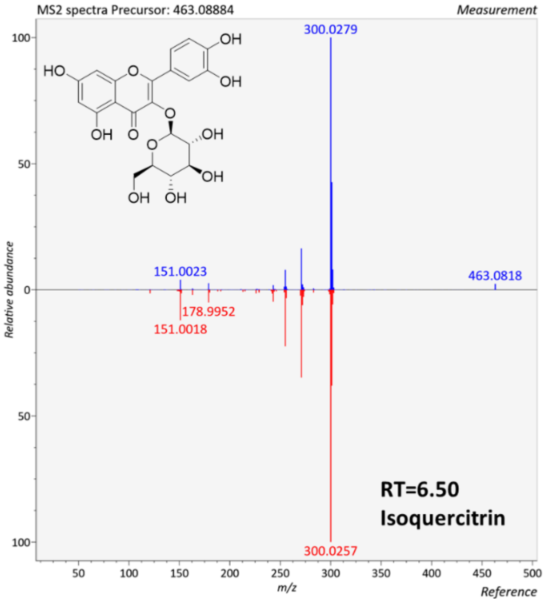
MS2 spectra Precursor: 463.08884
Measurement
300.0279
151.0023
463.0818
178.9952
151.0018
300.0257
RT=6.50
Isoquercitrin
Relative abundance
m/z
Reference

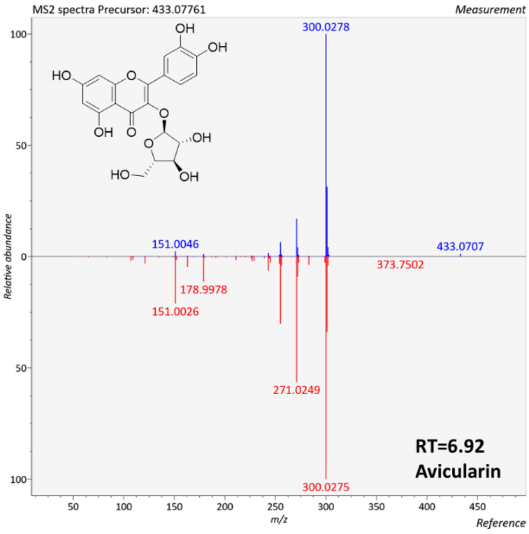
MS2 spectra Precursor: 433.07761
Measurement
300.0278
151.0046
433.0707
373.7502
178.9978
151.0026
271.0249
300.0275
RT=6.92
Avicularin
Relative abundance
m/z
Reference

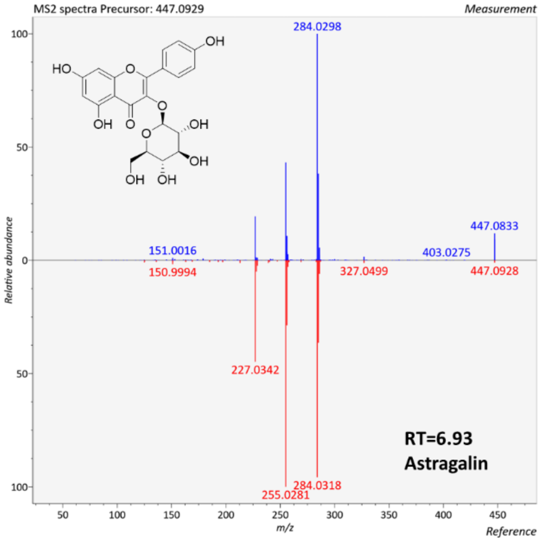
MS2 spectra Precursor: 447.0929
Measurement
284.0298
447.0833
151.0016
403.0275
150.9994
327.0499
447.0928
227.0342
255.0281
284.0318
RT=6.93
Astragalin
Relative abundance
m/z
Reference

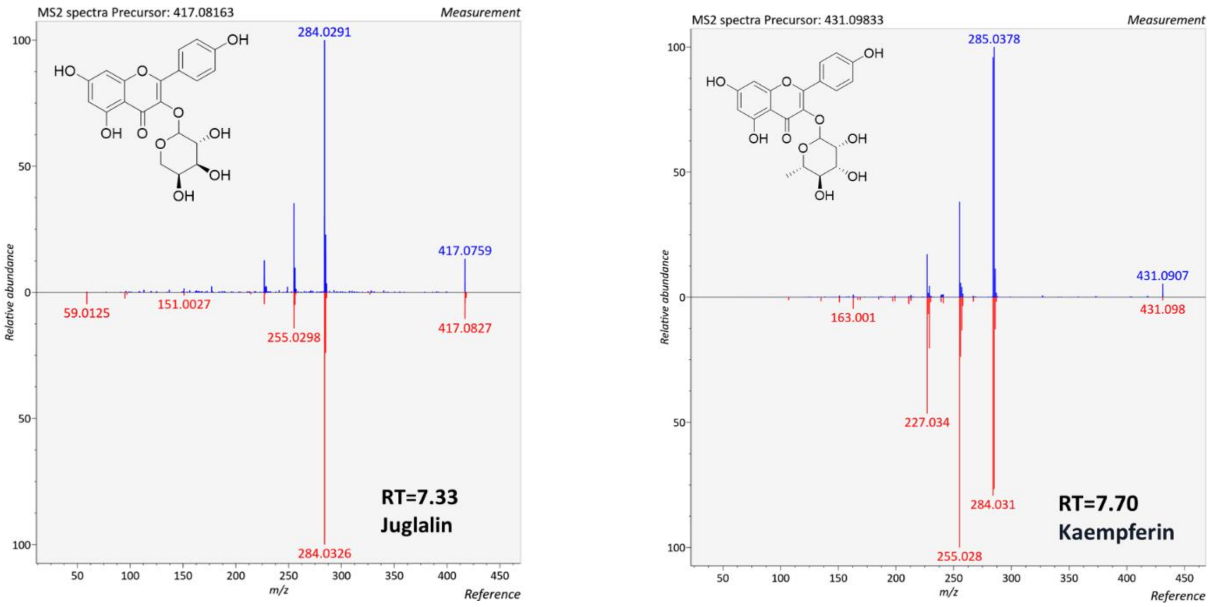

Figure S5. MS/MS spectra of the major compounds identified in polyphenols-enriched fraction (fraction B) obtained from BCHE extract. Experimental spectra (ES-) are reported in comparison with matched reference spectra.

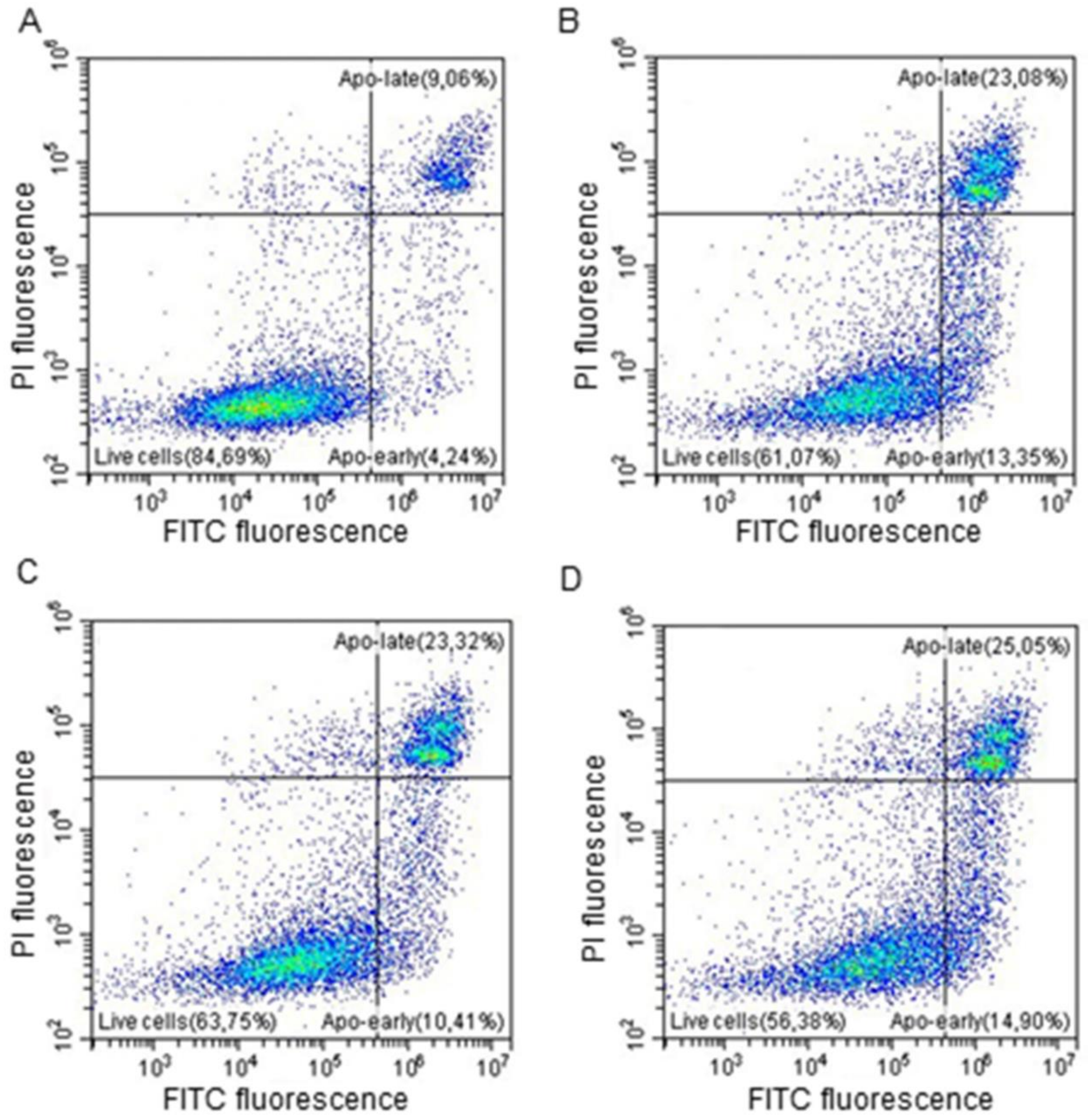

Figure S6. Apoptosis analysis by flow cytometry in Caco-2 cell line. Representative scatter plots show the distribution of annexin V- and PI-stained cells in control condition (panel **A**) and after treatment for 48 hours with 50 µg/mL of fractions B obtained from hydroalcoholic extracts of *Cinnamomum cassia* bark (CCHE) (panel **B**), *Cinnamomum zeylanicum* bark (CZHE) (panel **C**) and *Cinnamomum cassia* buds (BCHE) (panel **D**). The X-axis indicates annexin V–FITC fluorescence detected at 518 nm and the Y-axis indicates PI fluorescence detected at 620 nm. The lower left quadrant indicates live cells, the upper right quadrant indicates late apoptotic cells, the lower right quadrant indicates early apoptotic cells.

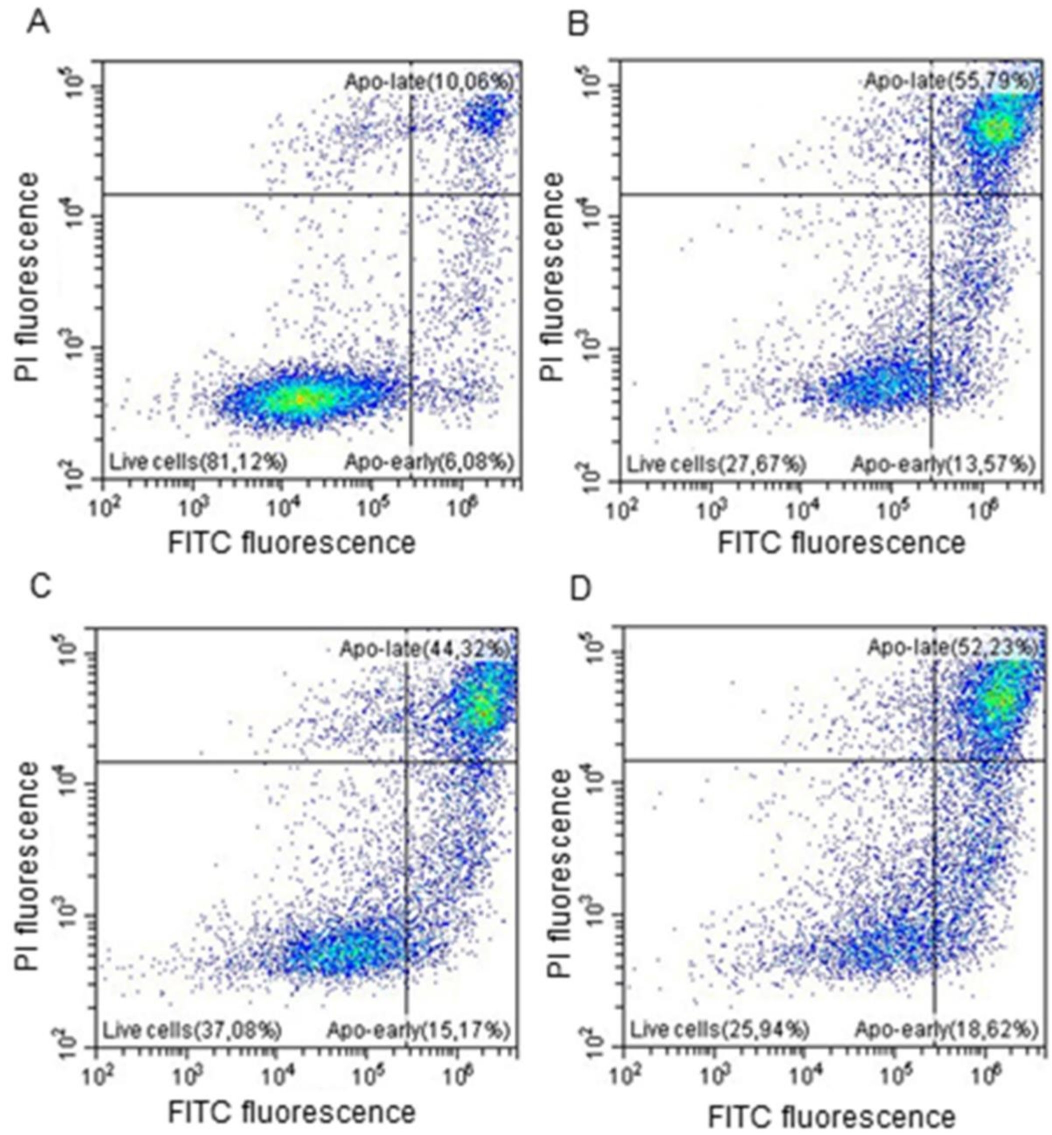

Figure S7. Apoptosis analysis by flow cytometry in E705 cell line. Representative scatter plots show the distribution of annexin V- and PI-stained cells in control condition (panel **A**) and after a 48 hours treatment with 50 µg/mL of fractions B obtained from hydroalcoholic extracts of *Cinnamomum cassia* bark (CCHE) (panel **B**), *Cinnamomum zeylanicum* bark (CZHE) (panel **C**) and *Cinnamomum cassia* buds (BCHE) (panel **D**). The X-axis indicates annexin V–FITC fluorescence detected at 518 nm and the Y-axis indicates PI fluorescence detected at 620 nm. The lower left quadrant indicates live cells, the upper right quadrant indicates late apoptotic cells, the lower right quadrant indicates early apoptotic cells.

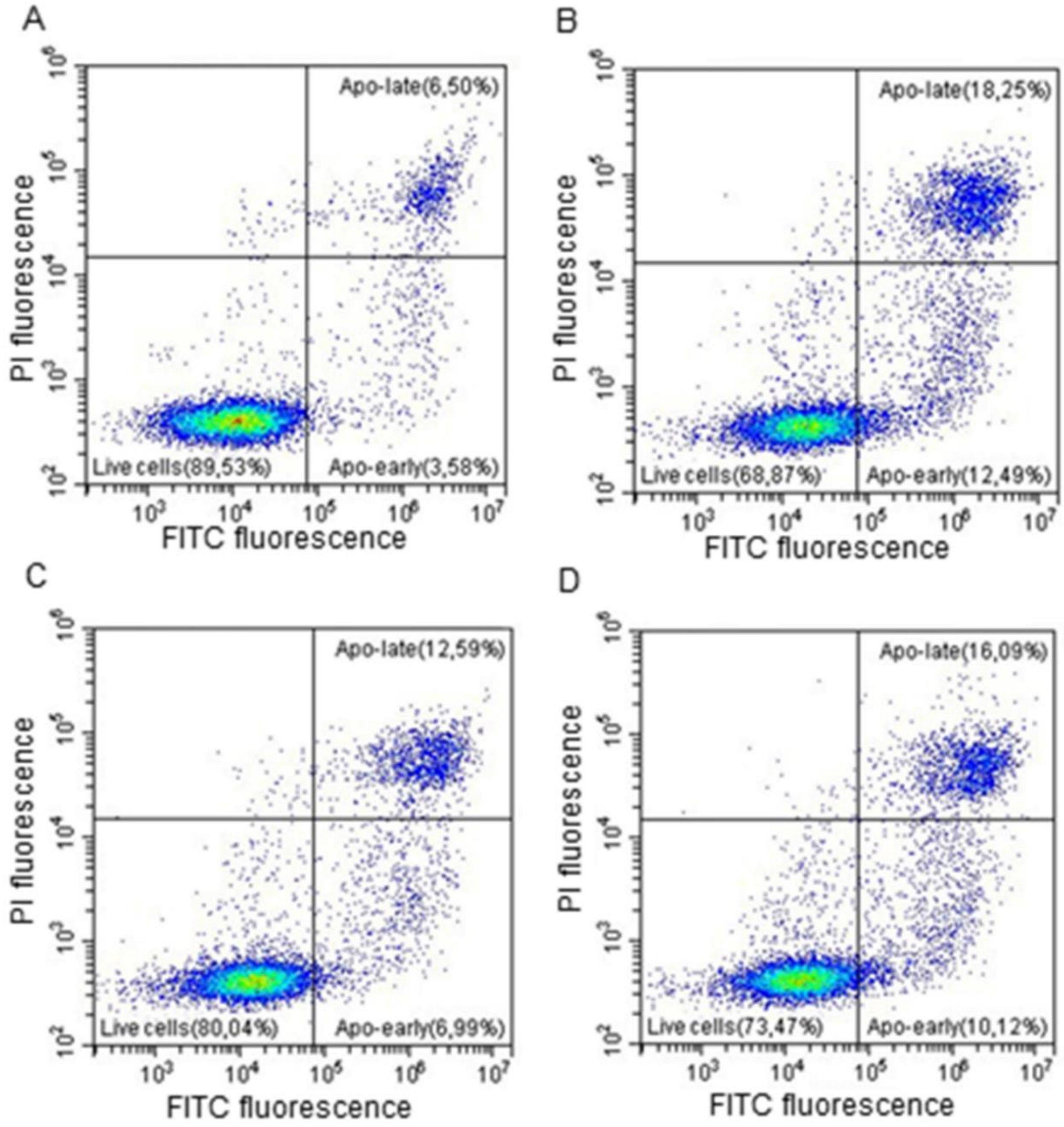

Figure S8. Apoptosis analysis by flow cytometry in SW480 cell line. Representative scatter plots show the distribution of annexin V- and PI-stained cells in control condition (panel **A**) and after 48 hours treatment for with 50 µg/mL of fractions B obtained from hydroalcoholic extracts of *Cinnamomum cassia* bark (CCHE) (panel **B**), *Cinnamomum zeylanicum* bark (CZHE) (panel **C**) and *Cinnamomum cassia* buds (BCHE) (panel **D**). The X-axis indicates annexin V–FITC fluorescence detected at 518 nm and the Y-axis indicates PI fluorescence detected at 620 nm. The lower left quadrant indicates live cells, the upper right quadrant indicates late apoptotic cells, the lower right quadrant indicates early apoptotic cells.
